# TriAIF-RWKV: A Physiology-Guided Spatiotemporal Framework for Robust Arterial Input Function Selection in CT Perfusion Imaging

**DOI:** 10.3390/e28090944

**Published:** 2026-08-22

**Authors:** Lei Lei, Yu Shen, Dawei Wang, Feng Xi, Yixin He, Chaochao Wang, Jiandong Liu

**Affiliations:** 1 College of Information Science and Engineering, Jiaxing University, Guangqiong Road, Jiaxing 314000, China; leilei@zjxu.edu.cn (L.L.); ccwang@zjxu.edu.cn (C.W.); jiandong.liu@zjxu.edu.cn (J.L.); 2 Provincial Key Laboratory of Multimodal Perceiving and Intelligent Systems, Jiaxing University, Guangqiong Road, Jiaxing 314000, China; 3 Department of Electronic Engineering, Nanjing University of Science and Technology, Xiaolingwei Road, Nanjing 210094, China; judy00196@njust.edu.cn (Y.S.); xifeng@njust.edu.cn (F.X.); 4 School of Electronics and Information, Northwestern Polytechnical University, Xi’an 710072, China; wangdw@nwpu.edu.cn

**Keywords:** arterial input function, CT perfusion, deep learning, RWKV network, acute ischemic stroke

## Abstract

Accurate delineation of infarct core and ischemic penumbra in acute ischemic stroke primarily relies on computed tomography perfusion (CTP), where the arterial input function (AIF) is essential for reliable perfusion quantification. However, reliable and fast AIF selection remains challenging in clinical practice due to noise, vascular heterogeneity, and inter-patient variability in bolus dynamics. In this study, we propose TriAIF-RWKV, a three-stage framework for robust and automated AIF extraction. Specifically, ACSANet is first employed for spatial vascular localization using axial and channel-aware attention mechanisms, thereby narrowing the candidate arterial region and reducing the AIF search space. Then, a Dilated-RWKV network is introduced to model temporal intensity dynamics from a global sequence perspective, allowing robust identification of AIF-consistent patterns. Finally, a physiology-informed scoring strategy is used to select the optimal AIF by evaluating baseline stability, peak enhancement, and washout characteristics. Extensive experiments on CTP datasets were conducted from multiple perspectives, including AIF waveform fidelity, perfusion parameter estimation, and lesion-level analysis. The results demonstrate that the proposed method achieved high agreement with expert-selected AIFs, with a global waveform PCC of 0.973, peak correlation of 0.942, and TTP correlation of 0.973 with a mean error of 0.923 s. Furthermore, the proposed method provides more consistent downstream perfusion quantification, achieving higher consistency of CTP-derived parameters and improved lesion-to-normal tissue discrimination compared with existing approaches. These results highlight its potential for reliable clinical perfusion assessment.

## 1. Introduction

Acute ischemic stroke accounts for approximately 80–85% of all stroke cases, contributing to millions of deaths and disability-adjusted life years annually [1]. Rapid evolution of ischemic injury confines effective intervention to the first several hours after symptom onset, during which accurate delineation of irreversibly infarct core and potentially salvageable penumbra is essential for guiding treatment eligibility, therapeutic strategy and prognosis [2]. In the acute setting, computed tomography perfusion (CTP) imaging can effectively distinguish infarct core from ischemic penumbra by quantitative analysis of perfusion parameters, including cerebral blood flow (CBF), cerebral blood volume (CBV), and transit time related metrics, and other quantitative hemodynamic indices [3]. These parameters provide a reliable assessment of tissue viability and hemodynamic impairment, thereby making CTP the most effective and widely accepted technique for stroke evaluation and treatment decision-making [4]. However, reliable perfusion parameters critically depend on accurate selection of the arterial input function (AIF) [5,6]. Small deviations in AIF can introduce profound bias in perfusion parameters [7], for example, an insufficient AIF peak amplitude may lead to overestimation of CBF, while inaccuracies in bolus arrival time or dispersion can distort transit time metrics, motivating the need for fast, reproducible, and accurate AIF selection in time-critical workflows [8,9].

Traditionally, AIF selection in clinical practice primarily relied on manual approaches performed by experienced operators [9,10]. Manual selection is straightforward and can provide a physiologically plausible reference curve, and is therefore commonly regarded as a practical reference standard. Nevertheless, it is inherently subjective and time-consuming, with strong dependence on operator expertise and limited reproducibility. To address these limitations, numerous automatic AIF selection methods have been proposed to improve the efficiency and consistency [11]. Mouridsen et al. [12] proposed a fully automatic AIF procedure based on cluster analysis. Although this method reduces manual intervention and improves efficiency, its reliability depends on the separability of arterial and non-arterial curves and may be degraded by noise, insufficient arterial voxels, or partial-volume effects. Subsequently, Denis et al. [13] introduced a heuristic screening step before K-means clustering, in which candidate arterial voxels were selected using characteristic enhancement features such as early tracer arrival, high peak intensity, steep upslope, and rapid washout. This improves interpretability and reduces the influence of irrelevant voxels. However, its performance is highly dependent on predefined thresholds and empirical parameter settings, which are unlikely to generalise well across cases with varying signal inhomogeneity, bolus delay, or atypical enhancement dynamics. To mitigate the dependence on heuristic thresholds, Zhu et al. [14] proposed a gamma-variate function fitting-based approach that explicitly models the physiological bolus passage process rather than selecting candidate arterial voxels solely based on predefined curve-feature thresholds. This method provides a convenient way to characterize AIFs. However, it may lose patient-specific temporal information.

The above limitations highlight the difficulty of achieving accurate and efficient AIF selection. This has motivated the use of machine learning and deep learning approaches, which can learn AIF-relevant spatial and temporal representations directly from data rather than relying solely on predefined thresholds, clustering assumptions or idealised curve models. Fan et al. [15] proposed a multi-stream 3D CNN to estimate AIF regions from perfusion images. While this approach demonstrated the potential of deep learning for automated vascular localization, its training typically relies on manually annotated AIF regions or expert-selected AIFs, which may inherit annotation bias and inter-observer variability. To avoid explicit voxel picking, de la Rosa et al. [6] proposed AIFNet, an end-to-end framework that estimates vascular functions directly from 4D perfusion series by predicting voxel-wise probability maps and computing weighted averages over candidate voxels. This approach eliminates manual voxel selection and improves robustness against noise. However, the probability-weighted averaging strategy may introduce mean-value bias and over-smoothing, potentially weakening patient-specific hemodynamic characteristics. Furthermore, AIFNet mainly focuses on vascular candidate localization, while the complex temporal dynamics of perfusion signals are not explicitly modelled, limiting its ability to capture subtle physiological variations.

In fact, many temporal modeling approaches [16,17,18,19,20] have demonstrated strong capabilities in capturing long-range dependencies in time-series data. Early long short-term memory (LSTM)-based approaches have been widely adopted for AIF temporal modeling due to their ability to capture long-term dependencies and dynamic variations in curves. Espinosa et al. employed an LSTM-based framework to model the temporal evolution of CTP enhancement signals and identify stable AIF candidates [21]. Huang et al. further utilized LSTM to capture temporal dependencies between AIF and tissue responses for AIF correction [22]. Similarly, Soltanpour et al. showed that LSTM can effectively model temporal dependencies in 4D CTP dynamic enhancement sequences [23]. Despite these advances, LSTM-based approaches are limited by sequential computation and fixed-dimensional hidden states, which may restrict the modeling of complex temporal dependencies in AIF dynamics, particularly under delayed bolus arrival or truncated acquisition. To address these limitations, temporal convolutional networks (TCNs) provide an alternative by leveraging dilated convolutions to efficiently capture multi-scale temporal dependencies [24]. Amado et al. demonstrated that TCN-based models could learn clinically meaningful temporal characteristics from 4D CTP curves [25]. Nevertheless, TCNs still rely on predefined convolutional receptive fields, and their ability to capture long-range temporal dependencies may be limited by network depth and dilation configurations. Transformer-based architectures further advanced temporal modeling by leveraging self-attention mechanisms. Although Transformer models have not been directly applied to AIF selection, their effectiveness in analysing CTP dynamic characteristics has recently been demonstrated. Luo et al. [26] developed a physics-informed Transformer framework that models the relationship between tissue enhancement curves and AIFs, enabling improved CTP parameter estimation through long-range temporal dependency learning. Nevertheless, the quadratic complexity of self-attention introduces substantial computational costs, limiting the efficiency of Transformer-based models for large-scale AIF screening. This motivates the development of more computationally efficient architectures capable of modeling long-range temporal dependencies. The Receptance Weighted Key Value (RWKV) architecture proposed by Peng et al. [27] achieves this goal by combining recurrent computation with attention-like long-range dependency learning. It achieves linear computational complexity while maintaining global temporal modeling capability, making it suitable for AIF identification by efficiently capturing both rapid contrast bolus dynamics and long-range enhancement patterns.

In addition to AIF curve quality, accurate localization of the arterial region is also critical for reliable CTP quantification. Recently, Winder et al. employed a CNN-based framework for voxel-wise arterial classification [28], while Saca et al. used a 3D U-Net to identify AIF-related arterial regions [29]. These studies demonstrated the feasibility of arterial localization for AIF extraction. However, these approaches mainly focus on identifying arterial regions based on local image features, without considering the surrounding vascular context or the physiological characteristics of arterial enhancement curves. Therefore, a more effective mechanism for modeling global spatial relationships is desirable. Axial Attention proposed by Wang et al. [30] provides an efficient solution by decomposing self-attention into directional operations, which is beneficial for modeling vascular contextual information in CTP images. However, spatial attention alone may overlook the varying importance of different feature channels. Therefore, the combination of axial spatial attention with channel attention can jointly enhance vascular spatial context and feature discrimination for accurate arterial localization.

Based on the above analysis, existing deep learning-based AIF extraction methods still have limitations in jointly modeling vascular spatial characteristics, temporal enhancement dynamics, and physiological constraints. Therefore, we propose TriAIF-RWKV, a unified three-stage framework integrating arterial localization, temporal modeling, and physiology-guided candidate selection for robust AIF extraction. Specifically, an Axial Channel Spatial Attention Network (ACSANet) is developed to identify candidate arterial regions by exploiting directional spatial characteristics of cerebral vessels. A Dilated RWKV (Dil-RWKV) module is then introduced to efficiently capture multi-scale contrast bolus dynamics by combining local temporal variations with long-range dependencies. Finally, a physiology-driven scoring strategy is employed to refine candidate selection based on clinically relevant AIF characteristics, including baseline stability, peak enhancement, and washout behavior.

## 2. Method

**Figure 1:**
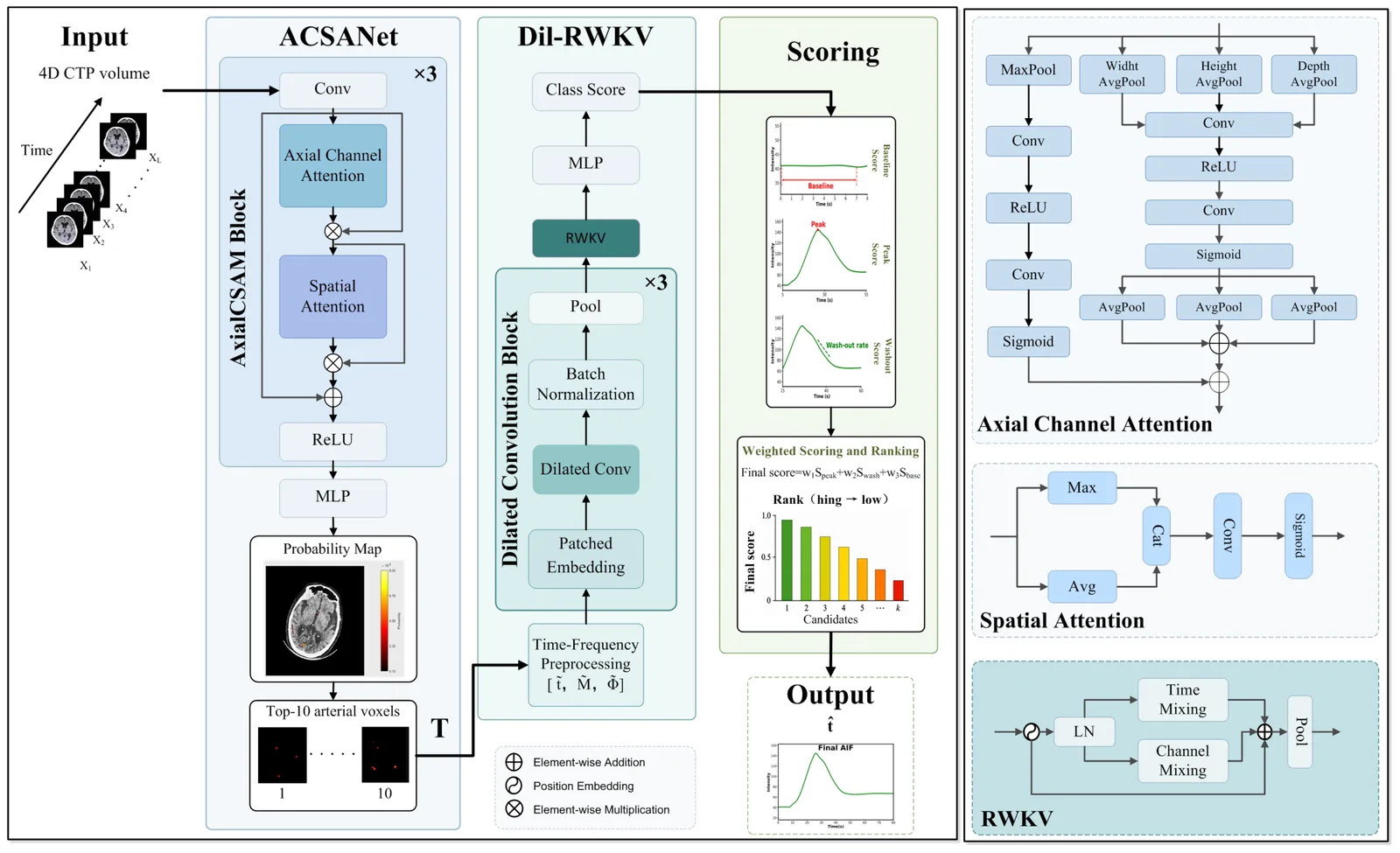
A schematic diagram illustrating the architecture of TriAIF-RWKV.

The proposed TriAIF-RWKV framework adopts a staged strategy for accurate AIF extraction, including ACSANet, Dil-RWKV network, and a multi-dimensional scoring mechanism. By progressively narrowing the candidate space from data-driven identification to physiology-informed evaluation, the framework ultimately derives a robust and representative AIF curve. The overall architecture is shown in Figure 1.

### 2.1. The Architecture of ACSANet

Reliable AIF extraction requires accurate localization of arterial voxels from CTP data. However, cerebral arteries exhibit complex spatial characteristics, while their appearance can be degraded by noise and partial-volume effects. ACSANet can effectively identify arterial candidate regions by leveraging axial spatial context and channel-wise feature refinement, enabling more accurate vascular localization. It adopts a hierarchical encoder architecture consisting of three cascaded feature extraction blocks followed by a voxel-wise prediction head. Each encoding block contains a convolutional layer, an Axial Channel Spatial Attention Module (AxialCSAM), and a nonlinear activation function. Through progressive feature abstraction, low-level intensity patterns are gradually transformed into high-level vascular representations, enabling robust localization of arterial structures. Given an input dynamic volumetric CTP sequence X, hierarchical spatial features are extracted as:

(1) $$F_{i}=\left\{\begin{array}{cc} \mathrm{ReLU}\left(A_{\mathrm{CSAM}}\left(\mathrm{Conv}\left(X\right)\right)\right), & i=1\\ \mathrm{ReLU}\left(A_{\mathrm{CSAM}}\left(\mathrm{Conv}\left(F_{i-1}\right)\right)\right), & i>1 \end{array}\right.$$

where $X\in R^{B\times L\times D\times H\times W}$ denotes the input 4D CTP sequence, with *B*, *L*, and (D,H,W) representing the batch size, number of temporal frames, and 3D spatial dimensions, respectively. The first convolutional block uses a 1×3×3 kernel to preserve temporal information while extracting spatial features, whereas the subsequent blocks employ 3×3×3 kernels for hierarchical volumetric feature learning. Dropout is applied after the first two encoding blocks to improve generalisation and reduce over-reliance on individual voxels. AxialCSAM is designed to enhance vascular-related representations by considering both directional spatial information and discriminative feature channels. Specifically, given an intermediate feature representation $Z\in R^{B\times L\times D\times H\times W}$, directional descriptors are first generated by aggregating features along the depth, height, and width axes:

(2) $$\begin{array}{c} \left\{\begin{array}{cc} Z_{d}\left(l,d\right) & =\frac{1}{HW}\sum_{h=1}^{H}\sum_{w=1}^{W}Z\left(l,d,h,w\right),\\ Z_{h}\left(l,h\right) & =\frac{1}{DW}\sum_{d=1}^{D}\sum_{w=1}^{W}Z\left(l,d,h,w\right),\\ Z_{w}\left(l,w\right) & =\frac{1}{DH}\sum_{d=1}^{D}\sum_{h=1}^{H}Z\left(l,d,h,w\right). \end{array}\right. \end{array}$$

These directional representations are used to generate axis-aware attention maps:

(3) $$\begin{array}{c} \left\{\begin{array}{c} A_{d}=\sigma \left(\mathrm{Conv}\left(f\left(Z_{d}\right)\right)\right),\\ A_{h}=\sigma \left(\mathrm{Conv}\left(f\left(Z_{h}\right)\right)\right),\\ A_{w}=\sigma \left(\mathrm{Conv}\left(f\left(Z_{w}\right)\right)\right). \end{array}\right. \end{array}$$

where f(·) denotes the channel compression operation implemented using a 1×1×1 convolution followed by ReLU activation, and σ represents the sigmoid function. The channel attention weight is obtained by combining direction-aware feature responses with global channel statistics:

(4) $$A_{c}=\left({\bar{A}}_{d}+{\bar{A}}_{h}+{\bar{A}}_{w}\right)/3+\sigma \left(\mathrm{Conv}(f(\mathrm{MaxPool}(Z)))\right),$$

where ${\bar{A}}_{d}$, ${\bar{A}}_{h}$ and ${\bar{A}}_{w}$ denote the average values of the corresponding directional attention maps.

In addition to channel refinement, spatial attention is employed to highlight arterial locations and suppress irrelevant tissue responses. The spatial attention map is calculated by concatenating average-pooled features $Z^{\mathrm{avg}}$ and max-pooled features $Z^{\max}$:

(5) $$A_{s}=\sigma \left(\mathrm{Conv}(\left[Z^{\mathrm{avg}},Z^{\max}\right])\right),$$

The final AxialCSAM output is formulated as $Y=Z+Z⊙A_{c}⊙A_{s}$, where ⊙ denotes element-wise multiplication. By integrating directional attention, channel discrimination, and spatial localization, AxialCSAM enhances arterial features while reducing interference from surrounding tissues. After the final encoding block, a 1×1×1 convolutional multilayer perceptron (MLP) projects the feature representation into a voxel-wise logit map S, which is normalised using spatial softmax:

(6) $$P\left(v\right)=\frac{\exp(S(v))}{\sum_{u\in \Omega }\exp\left(S\left(u\right)\right)},$$

where P(v) represents the probability of voxel *v* belonging to an arterial region, and Ω denotes the spatial domain of all voxels in the input image. The probability map provides a spatially constrained search space rather than directly averaging candidate voxels. Accordingly, the top-*k* voxels (*k* = 10) with the highest probabilities are selected as arterial candidates, and the curves extracted from local neighbourhood regions around these voxels are subsequently fed into the Dil-RWKV module for physiological temporal characterisation.

### 2.2. Dil-RWKV

Accurate AIF selection requires effective modeling of temporal contrast dynamics, as arterial enhancement exhibits both rapid local transitions and prolonged physiological evolution. Specifically, the early bolus arrival and peak enhancement involve sharp intensity variations, whereas the subsequent washout phase reflects long-range temporal dependencies associated with contrast dispersion and clearance. Therefore, we develop a Dilated-RWKV module to jointly capture local temporal variations and global physiological patterns by integrating dilated convolution-based temporal encoding with RWKV-based sequence modeling.

Given the candidate AIFs extracted from ACSANet, the input temporal matrix is denoted as $T\in R^{N\times L}$, where *N* represents the number of candidate curves. Dil-RWKV is used to classify the candidate curves according to their temporal quality. Specifically, the classifier contains three categories. Label 1 represents high-quality curves with smooth temporal evolution, low noise levels, and physiologically consistent perfusion characteristics. Label 2 represents intermediate-quality curves that generally follow physiological enhancement patterns but contain certain noise or deviations. Label 3 represents low-quality curves with significant noise or abnormal temporal characteristics, which are unsuitable for reliable AIF selection. The proposed Dil-RWKV consists of three stages: time–frequency representation learning, multi-scale temporal tokenization via dilated convolutions, and long-range dependency modeling by RWKV. The first stage enriches the temporal representation by incorporating complementary frequency-domain characteristics, while the second and third stages explicitly decouple local enhancement dynamics from global contrast evolution.

#### 2.2.1. Time–Frequency Representation

To enhance temporal representation, each candidate AIF is transformed into a joint time–frequency representation comprising time-domain, magnitude-spectrum, and phase-spectrum components. The temporal component preserves the dynamic evolution, whereas frequency-domain components provide complementary descriptions of temporal variations and signal dispersion. For each temporal signal $t\in R^{L}$, z-score normalization is first applied to obtain the normalized signal $\tilde{t}$. Subsequently, the discrete Fourier transform is applied along the temporal dimension from which the magnitude spectrum M and phase spectra Φ are extracted. Since the input signal is real-valued, only positive-frequency components are retained. The M is logarithmically transformed and normalized to reduce dynamic range, while the Φ is linearly scaled into the range of [0,1]. Finally, the temporal, magnitude, and phase representations are concatenated:

(7) $$D=\left[\tilde{t},\tilde{M},\tilde{\Phi }\right]\in R^{N\times L^{\prime }},$$

where $L^{\prime }$ represents the length of the combined temporal representation.

#### 2.2.2. Multi-Scale Temporal Tokenization via Dilated Convolutions

Dilated convolutions are introduced before sequence modeling to capture the multi-scale temporal characteristics of AIF dynamics. The combined representation D is first divided into non-overlapping temporal patches $d_{i}$ of length *M* along the temporal dimension. Then, each patch is mapped into a latent token through a modality-specific CNN encoder composed of residual blocks with dilated convolutional layers. Unlike conventional convolutional layers with limited receptive fields, dilated convolutions enlarge the temporal receptive field without increasing kernel size or reducing temporal resolution. By adopting different dilation rates, the encoder captures local temporal variations associated with bolus arrival, peak enhancement, and short-term intensity fluctuations. Subsequently, a global average pooling operation is applied to collapse the patch-length dimension, yielding a compact representation. As a result, each patch is represented by a *D*-dimensional token, independent of the original patch length *M*, and serves as the input to the subsequent RWKV-based sequence modeling stage.

#### 2.2.3. RWKV-Based Long-Range Temporal Modeling

In the proposed study, RWKV is employed to model long-range temporal dependencies among patch tokens with linear complexity, providing efficient sequence modeling for CTP analysis. For the input token sequence $Z^{k-1}$, the *k*-th RWKV block is formulated as:

(8) $$Z_{k}=Z_{k-1}+\mathrm{TimeMix}\left(\mathrm{LN}\left(Z_{k-1}\right)\right)+\mathrm{ChannelMix}\left(\mathrm{LN}\left(Z_{k-1}\right)\right),$$

where LN(·) denotes layer normalization. The TimeMix component aggregates historical temporal information through causal recurrent weighting, enabling modeling of physiological transitions from bolus arrival to peak enhancement and washout. The ChannelMix component further captures nonlinear interactions among feature dimensions to enhance the discrimination of different AIF patterns. Detailed formulations are referred to the original RWKV literature [31]. After multiple RWKV blocks, the final temporal representation is obtained by global aggregation:

(9) $$h=\frac{1}{L}\sum_{i=1}^{L}Z_{i},$$

and an MLP classifier maps the global representation h to the predicted class probability $\hat{y}$. The resulting classification provides an efficient temporal filtering mechanism, which is subsequently combined with physiology-driven scoring to determine the final AIF estimate.

### 2.3. Physiology-Guided Candidate Refinement and Final AIF Selection

**Figure 2:**
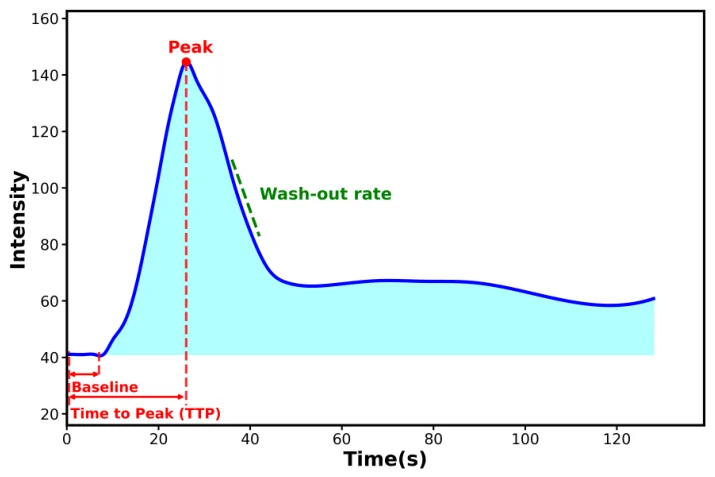
Schematic illustration of the physiological characteristics of an AIF curve.

To further incorporate physiological constraints and improve interpretability, a physiology-driven scoring strategy is introduced to rank candidates based on clinically meaningful AIF characteristics. For the candidate AIF set T extracted from ACSANet, Dil-RWKV predicts the temporal category probability for each candidate curve. Candidates classified as low-quality curves (label 3) are excluded, while the remaining AIF-like candidates are further evaluated using the proposed physiological scoring strategy. This design comprises three complementary scores, assessing baseline stability, first-pass enhancement, and post-peak decay characteristics of AIF curves. These characteristics are illustrated in Figure 2, representing baseline stability, peak enhancement, and washout consistency, respectively.

#### 2.3.1. Baseline Stability Score

Prior to contrast arrival, arterial signals should remain relatively stable. Large baseline fluctuations may indicate noise contamination, motion artefacts, or partial-volume effects. Therefore, the baseline stability score is defined as:

(10) $$S_{\mathrm{base}}=\frac{1}{1+\frac{1}{\eta }\sum_{i=1}^{b}\left|t_{i}\right|},$$

where *b* denotes the end point of the baseline phase and η is a normalization constant. A higher $S_{\mathrm{base}}$ indicates a more stable baseline.

#### 2.3.2. Peak Enhancement Score

The peak intensity $t_{p}$ reflects the maximum contrast enhancement during the first pass of the bolus and is closely related to arterial contrast concentration and partial-volume effects. In the absence of signal saturation, a higher peak amplitude generally indicates that the voxel is located closer to the centre of a major artery and is less affected by partial-volume contamination. Accordingly, the peak amplitude score is defined as

(11) $$S_{\mathrm{peak}}=\frac{t_{p}}{\eta }.$$

This score prioritises candidate curves with prominent arterial enhancement and well-defined first-pass characteristics within the candidate set.

#### 2.3.3. Washout Consistency Score

The physiological plausibility of the washout phase is critical for reliable estimation of the residue function, as it reflects contrast clearance and dispersion following the first-pass peak. A valid AIF is therefore expected to exhibit a smoothly decaying tail with minimal oscillations and a consistently negative decay trend. Let the washout segment be represented as $t_{\mathrm{tail}}=\left[t_{d},t_{d+1},\ldots ,t_{L}\right]$, where *d* denotes the start of the washout phase. The first difference $\Delta t_{i}$ and second discrete derivative $\Delta^{2}t_{i}$ are calculated, together with their variances $\sigma_{1}$ and $\sigma_{2}$, which quantify local fluctuations and curvature irregularities, respectively. In addition, linear regression is performed on the washout segment:

(12) $$t_{d+j}\approx a\;j+c,\;j=0,1,\ldots ,\left(L-d\right),$$

where *a* denotes the tail slope and *c* is the intercept of the fitted model. A negative slope indicates a physiologically consistent contrast clearance process. Accordingly, the washout consistency score is defined as:

(13) $$S_{\mathrm{wash}}=\frac{\max(0,-a)}{1+\sigma_{1}+\sigma_{2}}.$$

A higher $S_{\mathrm{wash}}$ indicates a smoother and more consistent washout pattern.

The overall physiological reliability score is then calculated as:

(14) $$S\left(t\right)=w_{1}S_{\mathrm{peak}}+w_{2}S_{\mathrm{wash}}+w_{3}S_{\mathrm{base}},$$

where $w_{1}$, $w_{2}$, and $w_{3}$ are weighting coefficients. Equal weights are assigned to the three components to avoid introducing empirical preference among different physiological characteristics. Finally, the candidate AIF with the highest physiological reliability score is selected as the final AIF:

(15) $$t^{*}=\arg\max_{t\in T_{c}}S\left(t\right),$$

where $T_{c}$ denotes the candidate set retained after Dil-RWKV temporal screening.

### 2.4. Loss Function and Training Strategy

The proposed framework was optimized using a stage-wise training strategy to decouple spatial arterial localization from temporal physiological characterization. In the first stage, ACSANet was trained to identify arterial candidate regions from 4D CTP volumes. Subsequently, the extracted candidate curves were manually labelled by the expert into three quality categories and used to train Dil-RWKV for temporal quality classification while keeping ACSANet parameters fixed. This strategy allows each module to be optimized according to its specific objective and improves training stability.

#### 2.4.1. Loss Function for ACSANet

ACSANet aims to localize arterial voxels by learning a voxel-wise probability map, from which the most probable arterial voxel is identified and its corresponding is extracted as the predicted AIF $\hat{t}$. Let $t^{*}$ denote the expert-annotated reference AIF. To optimize the localization accuracy indirectly through the extracted temporal profile, ACSANet is trained using a curve-level objective that jointly considers numerical agreement and temporal morphological consistency:

(16) $$L_{\mathrm{ACSANet}}=L_{\mathrm{MSE}}+\lambda L_{\mathrm{PCC}},$$

where λ is a weighting coefficient balancing amplitude fidelity and temporal shape consistency. The mean squared error (MSE) loss measures the amplitude difference between the predicted and reference AIF curves:

(17) $$L_{\mathrm{MSE}}=\frac{1}{L}\sum_{i=1}^{L}{\left(\hat{t}\left(i\right)-t^{*}\left(i\right)\right)}^{2}.$$

To further preserve the temporal morphology of contrast enhancement, a Pearson correlation coefficient (PCC)-based loss is introduced:

(18) $$L_{\mathrm{PCC}}=1-\frac{\sum_{i=1}^{L}\left(\hat{t}\left(i\right)-\bar{\hat{t}}\right)\left(t^{*}\left(i\right)-{\bar{t}}^{*}\right)}{\sqrt{\sum_{i=1}^{L}{\left(\hat{t}\left(i\right)-\bar{\hat{t}}\right)}^{2}}\sqrt{\sum_{i=1}^{L}{\left(t^{*}\left(i\right)-{\bar{t}}^{*}\right)}^{2}}}.$$

The PCC term encourages preservation of the temporal waveform characteristics, ensuring that the predicted AIF maintains physiologically consistent enhancement dynamics.

#### 2.4.2. Loss Function for Dil-RWKV

The Dil-RWKV module is trained to discriminate AIF-like candidates from unreliable curves using temporal characteristics. Given the predicted class probability vector $\hat{y}$, the classification objective is optimized using categorical cross-entropy loss:

(19) $$L_{\mathrm{Dil}\text{-}\mathrm{RWKV}}=-\frac{1}{N}\sum_{i=1}^{N}\sum_{c=1}^{C}y_{ic}\log\left({\hat{y}}_{ic}\right),$$

where C=3 represents the number of temporal quality categories, $y_{ic}$ denotes the expert-annotated one-hot encoded ground-truth label of category *c* for the *i*-th candidate curve, and ${\hat{y}}_{ic}$ represents the corresponding predicted probability. The ground-truth labels were manually assigned by the radiologist.

## 3. Experiments

To comprehensively evaluate the performance of the proposed AIF estimation method, we designed a multi-level experimental framework assessing its impact at the spatial localization, signal fidelity, and downstream clinical task levels.

### 3.1. Data and Preprocessing

This single-center retrospective study included a total of 176 patients with acute ischemic stroke, aged 53–86 years. All patients underwent dynamic CTP imaging as part of routine clinical assessment. The CTP examinations were acquired using two clinical CT scanners under routine workflow conditions. All datasets consisted of 16 axial slices and 40 dynamic phases, forming 4D perfusion volumes for AIF extraction, with a reconstructed slice thickness of 5 mm. The dataset included acquisitions from a Philips CT system and a Siemens CT system. The Philips scanner used dynamic axial scanning mode with an image matrix of 512 × 512 pixels, a tube voltage of 100 kVp, and a tube current of 303 mAs, while the Siemens scanner acquired images with a matrix size of 512 × 512 pixels, a tube voltage of 70 kVp, and a tube current of 616 mAs. The dataset was evaluated using a five-fold cross-validation strategy at the patient level. Specifically, the 176 patients were randomly divided into five non-overlapping subsets, consisting of 36, 35, 35, 35, and 35 patients, respectively. Data from the same patient were always assigned to the same subset to avoid data leakage. In each fold, four subsets were used for model training, while the remaining subset was used for validation. The final performance was reported by averaging the results across all five folds.

All CTP datasets underwent the same preprocessing pipeline, including contrast enhancement, noise reduction, and brain region segmentation. The reference AIFs were manually selected by a radiologist with 26 years of clinical experience based on established criteria, including anatomical localization within major cerebral arteries, early contrast arrival, high first-pass enhancement, minimal partial-volume effects, stable baseline signal, and smooth physiological washout. Physiological parameter estimation was performed using the MItalytics-CTP software (FISCA Healthcare, Nanjing, China). The Distributed Parameter (DP) model was selected for quantitative perfusion analysis because it is the kinetic model implemented in the MItalytics-CTP platform. Moreover, the DP model provides a comprehensive physiological description of capillary–tissue exchange and has been investigated in previous studies for quantitative perfusion analysis [32,33]. Importantly, the proposed AIF extraction method is independent of the kinetic model formulation and can theoretically be extended to other AIF-dependent perfusion models. The evaluated parameters included CBF, CBV, extraction fraction (E), mean transit time (MTT), permeability surface area product (PS), bolus arrival delay (tlag), and extravascular extracellular volume fraction (Ve).

Furthermore, to improve model generalisation and robustness to variations in contrast bolus timing, a physiology-guided temporal augmentation strategy was applied during training. Specifically, early-bolus and late-bolus augmentation were randomly selected to shift contrast enhancement dynamics to earlier or later time points, with a maximum displacement of five frames. The shifted sequences were completed by padding the terminal frames with the last acquired volume or the initial frames with the first acquired volume, respectively. The reference AIF curves were shifted accordingly to maintain temporal consistency between CTP sequences and their corresponding labels. Data augmentation was applied only to the training subsets, whereas the validation subsets remained unchanged.

### 3.2. Comparison Methods and Implementation Details

The proposed framework was compared with several representative AIF extraction approaches, including a traditional K-means clustering method [13], a fitting-based method [14], a CNN-based deep learning method [15], a U-Net-based method for arterial region localization [29], and AIFNet [6], a recent representative end-to-end AIF estimation framework. All experiments were conducted on the same workstation equipped with an NVIDIA GeForce RTX 4090 GPU (NVIDIA Corporation, Santa Clara, CA, USA), an Intel Xeon Silver 4210 CPU (Intel Corporation, Santa Clara, CA, USA) with 20 cores at 2.4 GHz, and 768 GB RAM. The learning-based models were trained and inferred using Python 3.10 with PyTorch 2.1.0 under Windows 11.

## 4. Results and Discussion

The performance of AIF estimation was evaluated from both qualitative and quantitative perspectives, following a hierarchical assessment strategy. Qualitative evaluation was conducted from two dimensions: anatomical rationality of candidate arterial voxels and physiological credibility of extracted AIFs. Quantitative evaluation was performed from two complementary perspectives: (i) the characteristic features of the estimated AIF curves, and (ii) the quality of perfusion parameter maps generated using the selected AIFs. This allows assessment of both AIF estimation fidelity and its impact on downstream perfusion analysis.

### 4.1. Qualitative Evaluation

**Figure 3:**
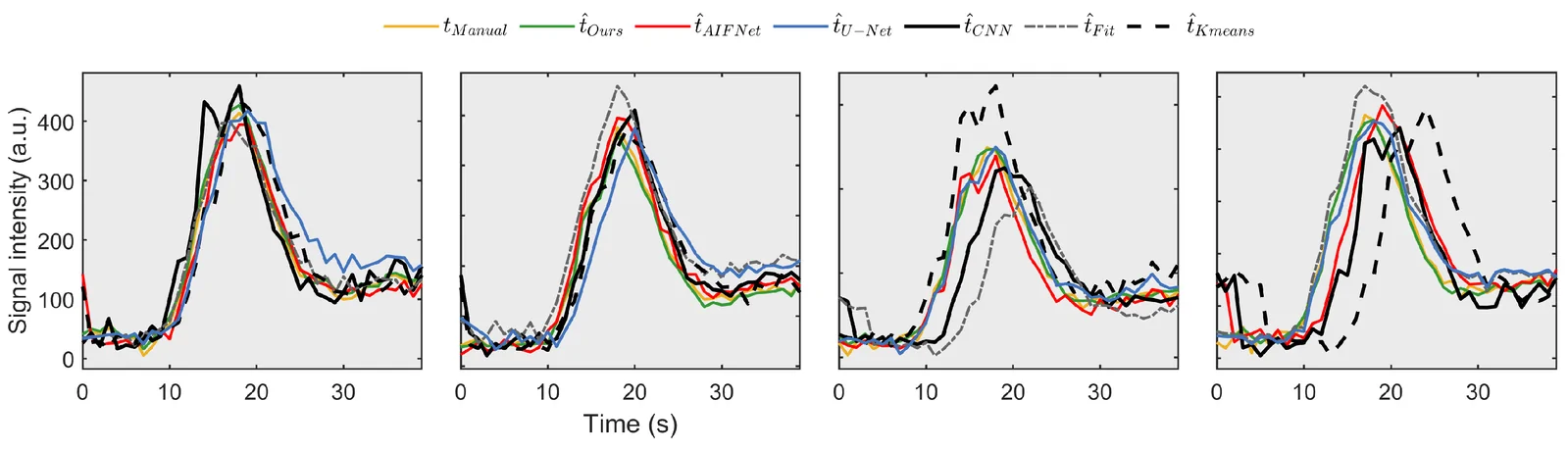
Comparison of AIF curve characteristics obtained by different methods.

**Figure 4:**
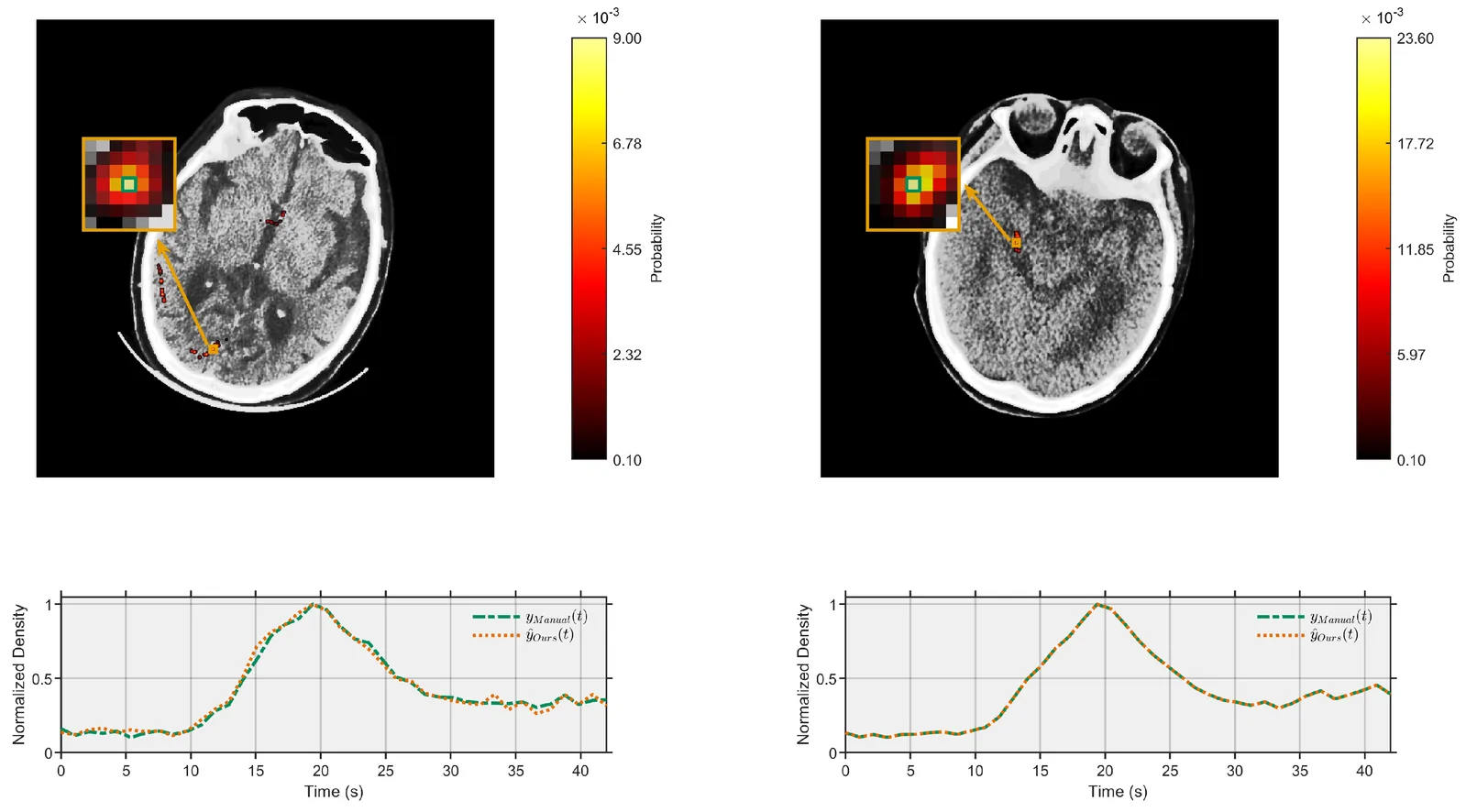
Comparison of AIF locations and curve profiles between the proposed method and manual selection. The green boxes indicate the arterial regions manually selected by the radiologist, while the arrow links the corresponding location to its enlarged view.

In terms of temporal curve characteristics, Figure 3 qualitatively compares the temporal profiles of AIFs derived from different algorithms against the manually annotated AIF curves. Visually, the AIF curve yielded by our proposed method exhibits superior alignment with the manual reference curve across all tested cases, with minimal deviations in overall temporal morphology relative to other comparative approaches. For spatial localization performance illustrated in Figure 4, the green framed region denotes the voxel position manually annotated by the radiologist. For samples with prominent, well-defined arterial signal features (right), the spatial coordinates of AIF voxels identified by our method achieve near-perfect consistency with expert manual delineation. Even for challenging cases featuring weak, indistinct arterial signal contrast (left), the voxel locations selected by our approach remain spatially proximal to the expert-marked region.

### 4.2. Curve Feature Evaluation

To evaluate the morphological and physiological reliability of the predicted AIFs, the estimated AIF $\hat{t}$ was quantitatively compared with the expert-annotated reference AIF $t^{*}$ for each subject. The assessment included global similarity, peak-amplitude accuracy, and time-to-peak (TTP) accuracy. The global similarity *r* was quantified using PCC, with its distribution summarized by the mean, standard deviation, and 5th–95th percentile range. For peak amplitude and TTP, the agreement was assessed using the PCC, and their absolute errors were also calculated and summarized as mean with standard deviation. TTP errors were reported in seconds.

Table 1 summarizes the quantitative comparison of the estimated AIF curves against the manually annotated reference in terms of global signal similarity, peak amplitude, and TTP accuracy. Overall, the proposed method achieved the best performance across all evaluated metrics. For global similarity, the proposed method obtained the highest mean PCC of 0.973, with the lowest standard deviation of 0.090. Its 5th–95th percentile range of 0.874–0.998 also indicates consistently high curve agreement across subjects. In comparison, the conventional Kmeans and fitting-based methods yielded substantially lower mean PCC values of 0.640 and 0.718, respectively, indicating a greater average loss of temporal information. Among the learning-based approaches, CNN and AIFNet achieved mean PCC values of 0.931 and 0.955, respectively, while the U-Net-based arterial localization method obtained a PCC of 0.874. Although U-Net improved upon the conventional methods by providing more reliable arterial localization, its performance remained lower than that of CNN and AIFNet, suggesting that spatial localization alone may not be sufficient to fully preserve the temporal characteristics of the AIF curve. Nevertheless, the proposed method further improved both the average similarity and the lower-percentile performance, indicating better preservation of AIF morphology, particularly in more challenging cases.

For peak-amplitude estimation, the proposed method achieved the strongest correlation with the manual reference, with a Pearson correlation coefficient of 0.942. This was higher than AIFNet, CNN, U-Net, fitting, and Kmeans, which achieved correlations of 0.877, 0.641, 0.667, and 0.450, respectively. The proposed method also produced the lowest peak-amplitude error, with a mean error of 17.00, compared with 39.20 for AIFNet, 37.68 for U-Net and over 40 for the remaining methods. This suggests that the proposed method provides a more reliable estimation of the arterial bolus peak intensity. For TTP estimation, the proposed method again achieved the best performance, with the highest correlation coefficient of 0.973 and the lowest mean error of 0.923 s. AIFNet and CNN also showed strong TTP correlations of 0.932 and 0.905, respectively, but their mean TTP errors were higher, at 1.538 s and 1.846 s. In contrast, Kmeans and fitting-based methods exhibited considerably larger TTP errors of 7.2 s and 6.4 s, reflecting their limited ability to localize the arterial peak accurately. Compared with AIFNet, the proposed method reduced the mean peak-amplitude error by approximately 56.6% and the mean TTP error by approximately 40.0%.

Given the competitive performance of AIFNet, additional statistical analysis was conducted to assess the significance of the observed differences. Specifically, paired comparisons were performed using the Wilcoxon signed-rank test. The 95% confidence intervals were estimated by bootstrap resampling, and *p* values were adjusted for multiple comparisons using the Benjamini–Hochberg FDR procedure. The same statistical analysis was applied to the subsequent quantitative comparisons. As summarized in Table 2, the proposed method demonstrated statistically significant improvements over AIFNet in global waveform similarity, peak enhancement estimation, and TTP accuracy after FDR correction ($p_{\mathrm{FDR}}<0.05$). The confidence intervals of the paired differences consistently excluded zero, indicating that the improvements were robust. These results indicate that the proposed method enables more stable and reliable AIF curve estimation.

### 4.3. Downstream Perfusion Parameter Evaluation

To investigate whether improved AIF estimation translates into more reliable downstream analysis, perfusion parameter maps derived from different AIF estimation methods were compared. All parameter maps were generated using the same kinetic modeling (DP) and post-processing framework, with only the input AIF varying across methods. The maps obtained using expert-selected AIFs were considered as the reference. The comparison was performed from multiple perspectives, including global agreement of perfusion parameter maps, regional consistency across clinically relevant tissue compartments, and the accuracy of perfusion-derived lesion delineation.

#### 4.3.1. Global Agreement of Perfusion Parameter Maps

Global agreement of perfusion parameter maps was first evaluated within the brain mask to investigate the overall impact of AIF on quantitative perfusion analysis. The agreement between automatic-AIF-derived maps and the manual-AIF-derived reference maps was assessed using three complementary voxel-wise metrics: PCC (*r*), normalized root-mean-square error (NRMSE), and bias. PCC quantified the preservation of spatial distribution patterns between the estimated and reference maps. NRMSE measured the magnitude of voxel-wise differences after normalization by the parameter scale. Bias was defined as the mean absolute voxel-wise difference from the reference map and reported in the original parameter units.

Table 3 summarizes the global agreement of perfusion parameter maps generated using different AIF estimation methods against the manual AIF reference. Overall, the proposed method achieved the most consistent agreement across multiple perfusion parameters, demonstrating improved preservation of spatial patterns, reduced voxel-wise deviations, and lower absolute bias. For the spatial agreement measured by PCC (*r*), all automatic AIF estimation methods showed relatively high consistency for flow-related parameters (CBF and CBV), whereas larger variations were observed for parameters more sensitive to temporal kinetics, such as E and PS. Compared with conventional clustering-based and curve-fitting approaches, learning-based methods achieved improved correlations, particularly for E, PS, and tlag. U-Net also showed strong performance in arterial localization, achieving correlations of 0.87 for CBF, 0.72 for E, and 0.90 for tlag. The proposed method obtained the highest correlation for most parameters, including CBF (0.88), CBV (0.87), MTT (0.78), PS (0.57), and Ve (0.82), indicating better preservation of the spatial distribution of the reference perfusion maps. For tlag, AIFNet achieved a slightly higher correlation (0.93) than the proposed method (0.92), suggesting that both approaches can accurately capture temporal arrival characteristics.

The NRMSE results further demonstrated the advantage of the proposed method in reducing voxel-wise discrepancies. Traditional methods exhibited larger errors, particularly for CBF, E, PS, and tlag, reflecting the sensitivity of quantitative perfusion parameters to inaccurate AIF estimation. In comparison, the proposed method achieved the lowest NRMSE across all evaluated parameters, with notable improvements for CBV (0.03), MTT (0.09), PS (0.05), and Ve (0.07). The bias analysis showed similar trends. Compared with other automatic AIF estimation approaches, the proposed method consistently reduced the absolute deviation from the manual-AIF-derived reference maps. In particular, substantial bias reductions were observed for CBF, MTT, and tlag, where inaccurate AIF scaling or temporal alignment can directly affect quantitative estimation. Among the learning-based methods, U-Net achieved relatively low NRMSE for several parameters, including CBF (0.07), E (0.08), and tlag (0.10), but remained less consistent across all metrics than the proposed method. In comparison, the proposed method achieved the lowest bias for CBF (9.01), E (1.48), MTT (0.32), and tlag (0.26), demonstrating improved quantitative reliability. Although FIT and CNN achieved relatively low bias for CBV and Ve, respectively, their lower spatial agreement and higher voxel-wise errors suggest that these improvements may not fully translate into consistent whole-map accuracy.

The statistical analysis was also performed between the proposed method and AIFNet in this experiment. As summarized in Table 4, the proposed method achieved statistically significant improvements in several perfusion parameters in terms of both spatial agreement (PCC) and quantitative accuracy (NRMSE). Specifically, significant improvements were observed for CBF, CBV, and MTT after FDR correction, indicating that the more accurate AIF estimation led to improved perfusion parameter recovery. For some parameters, such as tlag, the confidence intervals included zero and no statistically significant difference was observed, suggesting comparable rather than superior performance compared with AIFNet. Nevertheless, the proposed method achieved significant improvements in several key parameters while maintaining comparable performance in the remaining ones, demonstrating more consistent quantitative accuracy.

#### 4.3.2. Region-Wise Quantitative Agreement

We further evaluated the regional consistency of perfusion parameters across different tissue compartments, including the infarct core, ischemic penumbra, and normal tissue. The infarct core and ischemic penumbra were obtained from the clinical perfusion assessment, while normal tissue regions were manually selected from the contralateral healthy hemisphere. These predefined regions were fixed and consistently applied to all parameter maps. For each perfusion parameter, regional mean values were calculated, and the relative differences between automatic-AIF-derived and reference maps were summarized across subjects as mean with standard deviation. This regional analysis evaluated whether different AIF estimation methods could preserve clinically relevant perfusion characteristics and maintain the relative differences between pathological and normal tissues, beyond voxel-wise map similarity.

Table 5 reports regional differences. Overall, the proposed method achieved lower regional discrepancies for most perfusion parameters. In the infarct core region, conventional AIF selection methods exhibited relatively large deviations, particularly for E and PS, with the Kmeans method showing the largest discrepancies. This may be due to the lack of physiological constraints in Kmeans, which relies only on signal similarity and may not capture hemodynamic characteristics in hypoperfused tissue. Compared with other approaches, the proposed method achieved the lowest differences for CBF (0.26), CBV (0.11), E (0.22), MTT (0.27), tlag (0.18), and $V_{e}$ (0.49), demonstrating improved robustness in capturing altered hemodynamic characteristics within infarct regions. In the ischemic penumbra, where perfusion impairment is heterogeneous and intermediate, the proposed method also achieved the lowest relative differences for CBF (0.37), E (0.21), MTT (0.17), and PS (0.39), while maintaining comparable performance for the remaining parameters. Although other learning-based methods achieved lower discrepancies for individual parameters, their improvements were not consistent across all perfusion metrics. In normal-appearing tissue, the proposed method further reduced regional differences for most parameters, including CBF (0.25), E (0.52), MTT (0.23), tlag (0.27), and $V_{e}$ (0.26). Meanwhile, the proposed method showed significantly reduced regional discrepancies for several key perfusion parameters compared with AIFNet, particularly in the infarct core and penumbra. For the remaining parameters, the proposed method maintained comparable performance, indicating stable perfusion quantification across both ischemic and normal tissue regions. These findings demonstrate that the proposed method enables more reliable downstream perfusion quantification and better preserves clinically relevant perfusion differences across heterogeneous tissue compartments.

#### 4.3.3. Spatial Agreement of Perfusion-Derived Lesion Regions

**Figure 5:**
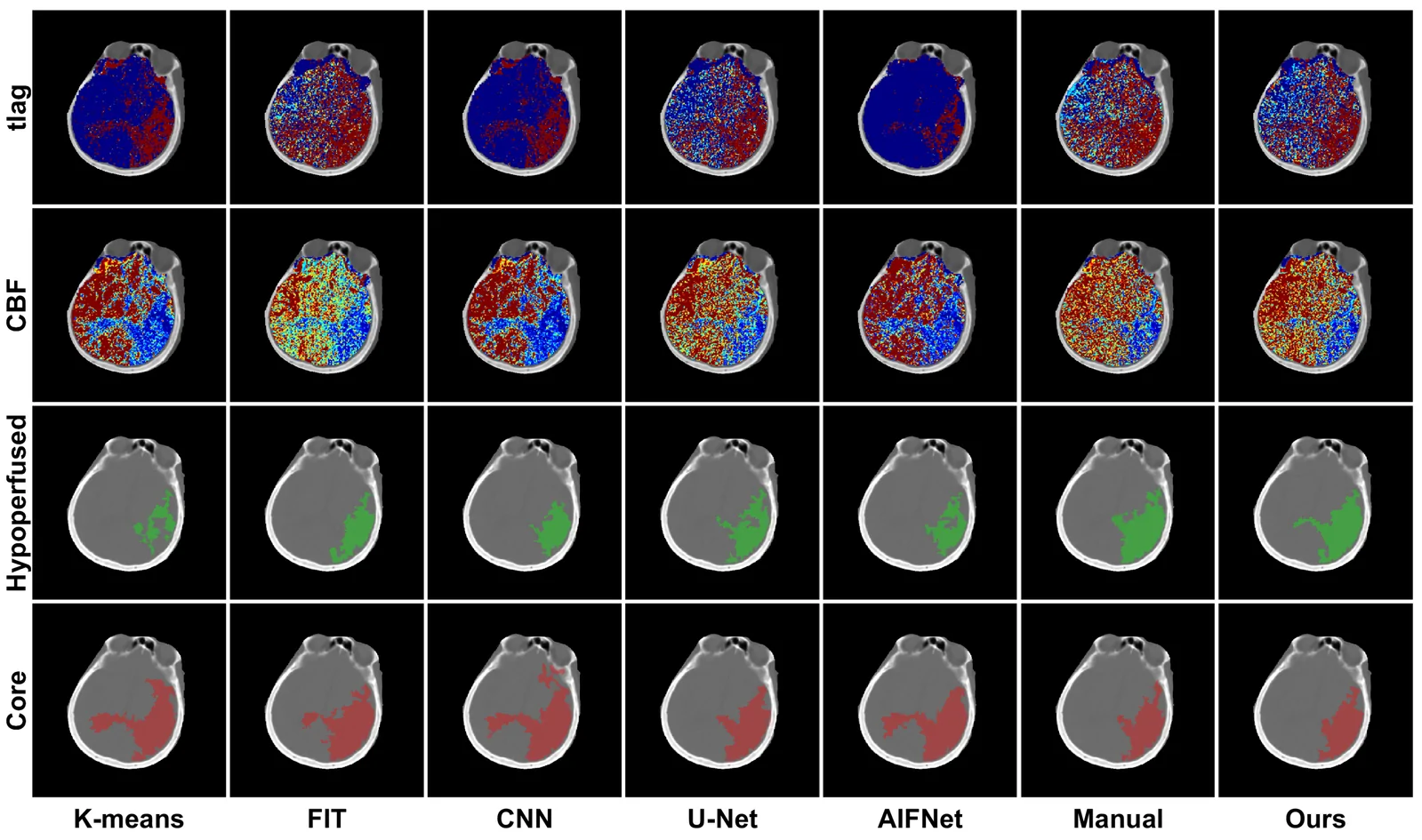
Estimated parameter maps and brain lesions for all the methods. The green and red regions in the third and fourth rows indicate the segmented hypoperfused and infarct core regions, respectively.

Spatial agreement of perfusion-derived lesion regions was evaluated to assess the impact of AIF estimation on lesion localization and boundary delineation. Infarct core regions were defined from relative CBF (rCBF) maps, where CBF values were normalized using the reference value from normally perfused tissue in the contralateral hemisphere to preserve voxel-wise relative contrast across subjects. A fixed threshold of 0.3 was applied to rCBF maps generated from all AIF methods for infarct core delineation. Similarly, penumbra regions were identified from tlag maps using a consistent threshold of 6 s across all methods. The resulting lesion masks were compared with the manual-AIF-derived reference masks. A representative case illustrating the lesion segmentation results derived from the CBF and tlag maps is shown in Figure 5.

Structural similarity index measure (SSIM) was used to evaluate structural agreement between lesion maps. Dice similarity coefficient and intersection over union (IoU) were used to quantify spatial overlap, while the 95th percentile Hausdorff distance (HD95) and average surface distance (ASD) were used to assess boundary discrepancies and spatial displacement. Precision and recall were additionally reported to characterize false-positive and false-negative errors. Together, these metrics provided a comprehensive evaluation of the spatial agreement of perfusion-derived lesion regions.

Table 6 summarizes the spatial agreement of perfusion-derived lesion regions obtained using different AIF estimation methods against the manual AIF reference. Overall, the proposed method achieved improved lesion localization and boundary consistency for both infarct core and hypoperfused regions. For infarct core delineation, the proposed method obtained the highest Dice (0.21), SSIM (0.88), and recall (0.57), together with the lowest HD95 (55.33) and ASD (25.15), indicating better agreement with the manual-AIF-derived reference, particularly in preserving lesion boundaries and reducing missed regions. U-Net achieved competitive performance in several overlap-related metrics, with a Dice of 0.18 and recall of 0.56, but showed larger boundary errors than the proposed method. Although AIFNet achieved a slightly higher IoU (0.14) and precision (0.15), the proposed method showed better overall spatial consistency. For hypoperfused regions, the proposed method consistently reduced boundary discrepancies, achieving the lowest HD95 (114.2) and ASD (31.74), while obtaining the highest Dice (0.10), SSIM (0.88), IoU (0.06), and precision (0.12). Compared with conventional AIF estimation methods, which exhibited larger localization errors and lower overlap, the proposed method demonstrated improved preservation of perfusion-derived lesion regions. These results suggest that more accurate AIF estimation can reduce error propagation in downstream lesion characterization and provide lesion maps closer to those derived from the manual AIF reference.

#### 4.3.4. Preservation of Lesion–Normal Tissue Perfusion Contrast

Lesion-to-normal tissue contrast was evaluated to quantify the discrimination between infarct regions and contralateral normal tissue on perfusion parameter maps. The infarct core regions were defined using DWI lesions as the reference standard, while contralateral normal tissue regions were selected from the corresponding healthy hemisphere. For each parameter, these predefined regions were used to compute the contrast-to-noise ratio (CNR) and effect size. CNR measures the difference in mean perfusion values between lesion and normal tissue relative to their within-region variability, while effect size quantifies the standardized difference between the two tissue distributions. Both metrics were calculated using the mean and standard deviation of CBF values within the predefined regions. Higher CNR and larger absolute effect size indicate better preservation of CBF contrast between infarct core and normal tissue.

Table 7 presents the CBF separability results for different AIF selection methods. Overall, the proposed method achieves the highest CNR (1.28) and effect size (0.81), indicating improved contrast and more pronounced statistical separation between infarcted and normal tissue compared with all competing approaches. In particular, it consistently outperforms traditional methods such as Kmeans and FIT, as well as learning-based baselines including CNN, U-Net and AIFNet. Statistical analysis further demonstrated that the improvements over AIFNet were significant after FDR correction ($p_{\mathrm{FDR}}<0.05$). These results suggest that more accurate AIF estimation contributes to improved lesion-to-normal tissue discriminability in derived perfusion maps.

### 4.4. Component-Wise Ablation Analysis

To investigate the contribution of each component in the proposed TriAIF-RWKV framework, an ablation study was performed. The effects of three key modules, including ACSANet, Dil-RWKV and the physiology-based scorer, were evaluated by progressively combining or removing individual components. All experiments were conducted using the same five-fold cross-validation protocol as the main experiments. As shown in Table 8, the progressive integration of each component consistently improved the accuracy of AIF estimation. The ACSANet-only model achieved an initial AIF estimation by localising potential arterial candidates, with a peak error of 34.5 and a TTP error of 1.86 s. After incorporating Dil-RWKV, the peak and TTP errors were reduced to 21.6 and 1.18 s, respectively, demonstrating the benefit of temporal enhancement modeling for distinguishing AIF-like curves. The physiology-based scorer further refined candidate selection by incorporating physiological curve characteristics, reducing the peak error and TTP error to 17.0 and 0.92 s in the final TriAIF-RWKV framework. These results confirm the complementary roles of spatial localization, temporal modeling, and physiological refinement in the proposed framework.

The component removal experiments further validated the contribution of each module. Removing ACSANet reduced the performance (Peak *r*: 0.84, Peak error: 31.0), confirming the importance of accurate spatial candidate localization. Without Dil-RWKV, the model showed degraded peak estimation (Peak *r*: 0.83, Peak error: 32.1), demonstrating the necessity of temporal dynamic modeling. Removing the physiology-based scorer also resulted in performance degradation (Peak error: 21.6 vs. 17.0 in the full model), indicating its role in physiological candidate refinement. Overall, the three components provide complementary functions for reliable AIF extraction.

To further evaluate the effectiveness of the proposed Dil-RWKV module, alternative temporal encoders, including LSTM and Transformer, were evaluated while keeping ACSANet and the physiology-based scorer unchanged. As shown in Table 9, Dil-RWKV achieved the best performance among all temporal modeling strategies, with a higher global PCC (0.973) and lower peak and TTP errors (17.00 and 0.923 s) compared with LSTM (0.962, 22.83, and 1.251 s) and Transformer (0.966, 21.47, and 1.162 s). This improvement can be attributed to the ability of Dilated-RWKV to effectively capture both local enhancement variations and global temporal dependencies in CTP time-intensity curves, leading to more accurate characterization of arterial enhancement dynamics.

### 4.5. Clinical Workflow Integration and Limitations

In clinical practice, the proposed TriAIF-RWKV framework is designed as an automated AIF selection module integrated into the existing CTP post-processing workflow rather than replacing the entire perfusion analysis pipeline. Given the dynamic CTP images, the framework automatically identifies arterial candidate regions, characterizes temporal enhancement patterns, and selects the most representative AIF curve. The selected AIF can be directly transferred to the existing kinetic modeling module for subsequent generation of quantitative perfusion maps. Therefore, the proposed framework can be incorporated into current CTP analysis platforms by replacing the manual AIF selection step while maintaining the existing downstream perfusion quantification procedure. Importantly, the proposed method is intended to assist rather than replace clinical decision-making. In practical deployment, the automatically generated AIF can serve as an objective reference for clinicians, while manual AIF selection remains available when necessary. This automated–manual dual-selection strategy provides flexibility for different clinical scenarios and preserves clinician control during the CTP interpretation process.

From the perspective of computational efficiency, the proposed framework introduces minimal additional computational burden to the overall CTP workflow. On the experimental workstation, TriAIF-RWKV required approximately 1–2 s to process a complete CTP volume (16 axial slices × 40 dynamic phases), including arterial candidate localization, temporal curve characterization, and physiology-guided candidate refinement. In comparison, the overall clinical CTP post-processing workflow typically involves multiple procedures which generally require several minutes. Therefore, the additional inference time introduced by the proposed method is negligible and is unlikely to affect clinical turnaround time.

Despite the promising performance, several limitations should be acknowledged. First, this study was conducted using a retrospective dataset from a single medical centre, and the sample size was relatively limited. Although the dataset included CTP examinations acquired using two different clinical CT scanners, validation on larger and more heterogeneous datasets collected from multiple centres and imaging protocols is still necessary to further establish the generalizability and robustness of the proposed framework. Future work will focus on collecting additional CTP data from broader clinical populations to further evaluate the performance of the proposed method under diverse clinical conditions. Second, the reference AIFs used in this study were obtained through expert manual selection, which may inevitably introduce subjective variability. Although the annotations were performed by an experienced radiologist following established physiological criteria, future studies involving multiple experts and inter-observer agreement analysis would provide a more comprehensive evaluation of reference AIF variability. Finally, the downstream perfusion evaluation in this study was performed using the DP model implemented. Although the DP model provides a comprehensive physiological representation of capillary–tissue exchange and has been validated in previous studies, different kinetic models may exhibit different sensitivities to AIF variations. Therefore, further studies using alternative kinetic modelling approaches are required to systematically evaluate the generalizability of the proposed AIF extraction method across different perfusion analysis frameworks.

## 5. Conclusions

This study presents TriAIF-RWKV, a three-stage framework for AIF selection that integrates spatial vascular localization, temporal curve characterization, and physiology-driven selection. Unlike conventional approaches that rely on heuristic rules, clustering stability, or parametric curve fitting, the proposed method explicitly separates spatial and temporal modeling, enabling more robust identification of arterial voxels and more accurate characterization of bolus dynamics. The ACSANet module improves arterial candidate localization by incorporating axial and channel-aware attention to preserve vascular continuity under noise and partial-volume effects, while the Dil-RWKV module captures non-stationary temporal dynamics with efficient long-range sequence modeling. A final physiology-driven scoring strategy further enforces clinically meaningful constraints, ensuring that selected AIFs exhibit consistent baseline stability, peak enhancement, and washout behavior.

Extensive evaluations demonstrate consistent improvements across multiple levels of analysis. At the signal level, the proposed method achieves superior agreement with expert AIFs in terms of global similarity and key hemodynamic features, including peak amplitude and time-to-peak accuracy. At the perfusion parameter level, it reduces voxel-wise error and bias across major CTP-derived maps (CBF, CBV, MTT, PS, and Ve), indicating improved stability of downstream kinetic modeling. More importantly, the proposed method improves lesion delineation performance in both infarct core and hypoperfused regions and enhances lesion-to-normal tissue separability as evidenced by higher CNR and effect size. These results collectively suggest that improved AIF selection not only enhances curve fidelity but also propagates to more accurate and spatially consistent perfusion quantification and clinically relevant lesion characterization.

## Figures and Tables

**Table 1:** Comparison of global curve similarity, peak intensity, and TTP metrics.

$t_{\mathrm{Manual}}$ vs.	Global		Peak		TTP	
	*r* ↑	*r* (P5^th^, P95^th^ ↑)	*r* ↑	Error ↓	*r* ↑	Error [s] ↓
${\hat{t}}_{Kmeans}$	0.640 (0.244)	(0.398, 0.976)	0.450	45.35 (64.72)	0.443	7.200 (6.573)
${\hat{t}}_{Fit}$	0.718 (0.175)	(0.464, 0.886)	0.667	41.15 (66.29)	0.560	6.400 (4.561)
${\hat{t}}_{CNN}$	0.931 (0.128)	(0.613, 0.997)	0.641	42.12 (78.11)	0.905	1.846 (3.246)
${\hat{t}}_{U\text{-}Net}$	0.874 (0.098)	(0.709, 0.982)	0.623	37.68 (64.60)	0.799	5.60 (3.651)
${\hat{t}}_{AIFNet}$	0.955 (0.121)	(0.772, 0.998)	0.877	39.20 (36.59)	0.932	1.538 (3.409)
${\hat{t}}_{Ours}$	**0.973 (0.090)**	**(0.874, 0.998)**	**0.942**	**17.00 (19.83)**	**0.973**	**0.923 (2.058)**

Note: ↑ indicates that a higher value is better, whereas ↓ indicates that a lower value is better. Bold values indicate better performance. The same notation applies to the tables below.

**Table 2:** Statistical comparison between the proposed method and AIFNet.

Comparison	Global (*r*)	Peak		TTP	
		*r*	Error	*r*	Error [s]
95% CI	[0.012, 0.025]	[0.041, 0.083]	[−30.5, −15.6]	[0.021, 0.059]	[−0.91, −0.32]
*p*	0.001	<0.001	<0.001	<0.001	0.002
$p_{\mathrm{fdr}}$	0.003	<0.001	<0.001	<0.001	0.004

**Table 3:** Global comparison of CTP-derived physiological parameters under different AIF methods vs. Manual AIF.

Metric		CBF	CBV	E	MTT	PS	tlag	Ve
	Method							
**r ↑**								
	K-means	0.81 (0.07)	0.76 (0.17)	0.21 (0.10)	0.63 (0.20)	0.12 (0.08)	0.43 (0.16)	0.69 (0.14)
	FIT	0.83 (0.06)	0.85 (0.08)	0.51 (0.11)	0.65 (0.12)	0.41 (0.17)	0.67 (0.13)	0.72 (0.13)
	CNN	0.81 (0.06)	0.84 (0.13)	0.55 (0.12)	0.65 (0.14)	0.48 (0.18)	0.66 (0.13)	0.72 (0.14)
	U-Net	0.87 (0.04)	0.83 (0.12)	0.72 (0.15)	0.77 (0.17)	0.48 (0.18)	0.90 (0.06)	0.80 (0.10)
	AIFNet	0.87 (0.05)	0.86 (0.08)	**0.76** (0.12)	0.77 (0.16)	0.56 (0.20)	**0.93 (0.07)**	0.80 (0.12)
	Ours	**0.88 (0.07)**	**0.87 (0.08)**	**0.76 (0.15)**	**0.78 (0.15)**	**0.57 (0.18)**	0.92 (0.04)	**0.82 (0.11)**
**NRMSE ↓**								
	K-means	0.41 (0.18)	0.12 (0.06)	0.18 (0.05)	0.17 (0.06)	0.16 (0.07)	0.22 (0.05)	0.15 (0.05)
	FIT	0.37 (0.18)	0.09 (0.04)	0.14 (0.04)	0.15 (0.05)	0.12 (0.06)	0.16 (0.04)	0.12 (0.04)
	CNN	0.29 (0.14)	0.08 (0.04)	0.12 (0.03)	0.14 (0.05)	0.10 (0.05)	0.15 (0.03)	0.11 (0.04)
	U-Net	0.07 (0.02)	0.11 (0.04)	0.08 (0.02)	0.11 (0.04)	0.09 (0.06)	0.10 (0.03)	0.08 (0.03)
	AIFNet	0.07 (0.03)	0.05 (0.02)	0.10 (0.03)	0.12 (0.03)	0.07 (0.04)	0.10 (0.03)	0.09 (0.03)
	Ours	**0.06 (0.02)**	**0.03 (0.01)**	**0.08 (0.02)**	**0.09 (0.02)**	**0.05 (0.02)**	**0.08 (0.02)**	**0.07 (0.02)**
**Bias ↓**								
	K-means	68.15 (23.81)	0.46 (0.18)	2.26 (0.86)	0.55 (0.2)	1.65 (0.63)	0.46 (0.17)	3.56 (1.24)
	FIT	43.64 (17.16)	**0.12 (0.05)**	2.03 (0.71)	0.54 (0.19)	**1.44 (0.51)**	0.38 (0.15)	2.2 (0.88)
	CNN	58.67 (23.03)	0.28 (0.08)	2.06 (0.75)	0.53 (0.2)	1.51 (0.52)	0.38 (0.15)	**1.85 (0.75)**
	U-Net	10.03 (2.25)	0.89 (0.16)	1.53 (0.45)	0.37 (0.15)	3.39 (2.11)	0.28 (0.11)	2.10 (0.78)
	AIFNet	9.94 (1.79)	0.44 (0.13)	1.5 (0.54)	0.37 (0.21)	3.24 (3.25)	0.41 (0.15)	3.11 (1.16)
	Ours	**9.01 (2.2)**	0.42 (0.07)	**1.48 (0.43)**	**0.32 (0.12)**	2.64 (1.94)	**0.26 (0.13)**	2.94 (1.06)

**Table 4:** Statistical comparison of perfusion parameter estimation between the proposed method and AIFNet.

Metric	Statistic	CBF	CBV	E	MTT	PS	tlag
**r**	95% CI	[0.003, 0.021]	[0.005, 0.025]	[−0.003, 0.018]	[0.008, 0.029]	[−0.002, 0.019]	[−0.014, 0.006]
	*p*	0.018	0.009	0.084	0.006	0.071	0.264
	$p_{\mathrm{FDR}}$	0.031	0.018	0.097	0.012	0.084	0.271
**NRMSE**	95% CI	[−0.035, −0.008]	[−0.031, −0.006]	[−0.026, −0.002]	[−0.041, −0.009]	[−0.028, −0.003]	[−0.019, 0.007]
	*p*	0.005	0.013	0.032	0.003	0.024	0.286
	$p_{\mathrm{FDR}}$	0.009	0.021	0.041	0.006	0.034	0.312

**Table 5:** Region-wise comparison of CTP perfusion parameters from different AIF estimation methods.

ROI	Method	CBF	CBV	E	MTT	PS	tlag	$V_{e}$
Core necrosis	K-means	0.77 (0.07)	0.57 (0.03)	0.98 (0.02)	0.76 (0.02)	0.95 (0.05)	0.57 (0.28)	0.93 (0.05)
	FIT	0.36 (0.18)	0.43 (0.08)	0.64 (0.23)	0.64 (0.16)	0.67 (0.18)	0.38 (0.09)	0.64 (0.10)
	CNN	0.56 (0.15)	0.44 (0.08)	0.74 (0.17)	0.60 (0.17)	0.65 (0.19)	0.32 (0.12)	0.68 (0.11)
	U-Net	0.32 (0.15)	0.65 (0.05)	**0.20 (0.13)**	0.34 (0.10)	0.67 (0.12)	0.26 (0.14)	0.52 (0.15)
	AIFNet	0.29 (0.18)	0.36 (0.07)	0.23 (0.14)	0.31 (0.11)	**0.50 (0.20)**	0.19 (0.10)	0.69 (0.10)
	Ours	0.26 (0.14) *	0.11 (0.07) *	0.22 (0.13)	**0.27 (0.08)**	**0.50 (0.20)**	**0.18 (0.09)**	0.49 (0.14) *
Penumbra	K-means	0.70 (0.19)	0.57 (0.09)	0.86 (0.28)	0.73 (0.11)	0.84 (0.23)	0.59 (0.30)	0.88 (0.07)
	FIT	0.51 (0.30)	0.41 (0.14)	0.65 (0.32)	0.60 (0.19)	0.62 (0.22)	0.34 (0.25)	**0.53 (0.18)**
	CNN	0.48 (0.23)	**0.19 (0.18)**	0.61 (0.31)	0.58 (0.24)	0.65 (0.22)	0.34 (0.25)	0.65 (0.16)
	U-Net	0.38 (0.25)	0.68 (0.06)	0.26 (0.12)	0.35 (0.12)	0.57 (0.24)	**0.28 (0.19)**	0.57 (0.15)
	AIFNet	0.43 (0.31)	0.48 (0.08)	0.24 (0.13)	0.23 (0.15)	0.47 (0.22)	0.32 (0.16)	0.72 (0.10)
	Ours	0.37 (0.27) *	0.45 (0.12)	**0.21 (0.16)**	0.17 (0.14) *	0.39 (0.28) *	0.39 (0.10)	0.73 (0.11)
Normal tissue	K-means	0.74 (0.15)	0.46 (0.20)	0.95 (0.17)	0.77 (0.18)	0.94 (0.11)	0.86 (0.24)	0.79 (0.14)
	FIT	0.62 (0.18)	**0.20 (0.23)**	0.88 (0.20)	0.79 (0.16)	0.82 (0.20)	0.79 (0.22)	0.63 (0.18)
	CNN	0.68 (0.19)	0.41 (0.17)	0.89 (0.19)	0.80 (0.17)	0.82 (0.13)	0.77 (0.25)	0.57 (0.18)
	U-Net	0.29 (0.20)	0.69 (0.13)	0.61 (0.25)	0.25 (0.23)	0.53 (0.23)	0.30 (0.23)	0.36 (0.18)
	AIFNet	0.26 (0.18)	0.52 (0.12)	0.54 (0.25)	0.31 (0.21)	0.44 (0.27)	0.40 (0.20)	0.40 (0.18)
	Ours	**0.25 (0.18)**	0.53 (0.12)	**0.52 (0.25)**	0.23 (0.23) *	**0.42 (0.23)**	0.27 (0.21) *	0.26 (0.18) *

Note: * indicates a statistically significant difference between the proposed method and AIFNet after FDR correction ($p_{\mathrm{FDR}}<0.05$).

**Table 6:** Spatial agreement of perfusion-derived lesion regions with the manual AIF reference.

	Core						Hypoperfused					
Method	HD95 ↓	Dice ↑	SSIM ↑	IoU ↑	Prec./Rec. ↑	ASD ↓	HD95 ↓	Dice ↑	SSIM ↑	IoU ↑	Prec./Rec. ↑	ASD ↓
K-means	68.49 (33.32)	0.05 (0.10)	0.82 (0.04)	0.03 (0.06)	0.06 (0.12)/0.06 (0.11)	28.80 (24.69)	127.4 (42.22)	0.06 (0.12)	0.82 (0.04)	0.04 (0.08)	0.05 (0.12)/0.21 (0.34)	41.99 (22.16)
FIT	59.97 (32.72)	0.17 (0.20)	0.83 (0.04)	0.11 (0.13)	0.12 (0.15)/0.40 (0.41)	29.45 (22.31)	142.6 (29.57)	0.09 (0.13)	0.83 (0.04)	0.05 (0.08)	**0.12** (0.18)/0.08 (0.13)	53.47 (25.76)
CNN	60.47 (33.98)	0.17 (0.20)	0.83 (0.04)	0.10 (0.13)	0.12 (0.16)/0.38 (0.39)	25.70 (24.15)	139.1 (52.56)	0.09 (0.16)	0.84 (0.03)	0.05 (0.11)	0.08 (0.16)/0.10 (0.17)	73.40 (46.44)
U-Net	67.34 (33.85)	0.18 (0.20)	0.87 (0.04)	0.12 (0.13)	0.12 (0.14)/0.56 (0.49)	29.02 (24.81)	141.9 (25.72)	0.04 (0.05)	0.87 (0.03)	0.02 (0.03)	0.03 (0.03)/0.17 (0.23)	33.87 (7.86)
AIFNet	59.47 (33.92)	0.19 (0.20)	0.87 (0.04)	**0.14 (0.16)**	**0.15 (0.18)**/0.45 (0.45)	25.51 (23.76)	126.0 (59.98)	0.06 (0.05)	0.87 (0.03)	0.03 (0.03)	0.03 (0.03)/**0.41 (0.36)**	36.92 (11.93)
Ours	**55.33 (21.97)**	**0.21 (0.24)**	**0.88 (0.04)**	0.12 (0.13)	0.12 (0.13)/**0.57 (0.49)**	**25.15 (21.50)**	**114.2 (36.30)**	**0.10 (0.17)**	**0.88 (0.03)**	**0.06 (0.12)**	**0.12 (0.19)**/0.10 (0.16)	**31.74 (12.29)**

**Table 7:** Lesion–normal tissue contrast preservation.

Method	CNR ↑	Effect Size ↑
K-means	1.15 (0.72)	0.58 (0.24)
FIT	1.15 (0.72)	0.69 (0.31)
CNN	1.21 (0.66)	0.65 (0.26)
U-Net	1.16 (0.68)	0.62 (0.25)
AIFNet	1.22 (0.71)	0.75 (0.34)
**Ours**	1.28 (0.67) *	0.81 (0.35) *

Note: * indicates a statistically significant.

**Table 8:** Ablation study of individual components in the proposed TriAIF-RWKV framework.

ACSANet	Dil-RWKV	Score	Global	Peak		TTP	
			*r* ↑	*r* ↑	Error ↓	*r* ↑	Error (s) ↓
✓	–	–	0.95	0.81	34.5	0.90	1.86
✓	✓	–	0.97	0.92	21.6	0.96	1.18
–	✓	✓	0.93	0.84	31.0	0.90	1.65
✓	–	✓	0.95	0.83	32.1	0.91	1.72
✓	✓	✓	**0.97**	**0.94**	**17.0**	**0.97**	**0.92**

Note: ‘✓’ indicates that the corresponding module is included, whereas ‘–’ indicates that it is not included.

**Table 9:** Comparison of different temporal modeling strategies.

Temporal Encoder	Global	Peak		TTP	
	*r* ↑	*r* ↑	Error ↓	*r* ↑	Error (s) ↓
LSTM	0.962	0.914	22.83	0.950	1.251
Transformer	0.966	0.923	21.47	0.957	1.162
Dil-RWKV	**0.973**	**0.942**	**17.00**	**0.973**	**0.923**

## Data Availability

The datasets generated during and/or analyzed during the current study are available from the corresponding author upon reasonable request.
